# Comparative Analysis of Thyroid Function Tests in Neonatal Intensive Care Unit Patients and Healthy Newborns

**DOI:** 10.7759/cureus.99542

**Published:** 2025-12-18

**Authors:** Gozde Gurpinar, Resat Gurpinar

**Affiliations:** 1 Department of Pediatric Endocrinology, Bagcılar Research and Training Hospital, Istanbul, TUR; 2 Department of Neonatology, Sisli Memorial Hospital, Istanbul, TUR; 3 Department of Pediatrics, Istanbul Arel University, Istanbul, TUR

**Keywords:** cesarean section, neonatal hypothyroidism, povidone-iodine, prematurity, thyroid screening, transient hypothyroxinemia

## Abstract

This study investigated the relationship between thyroid function and key perinatal factors, including mode of delivery, gestational age, and clinical condition, by comparing 152 neonates hospitalized in the neonatal intensive care unit (NICU) with 50 healthy newborns. Thyroid function tests (capillary thyroid-stimulating hormone (cTSH), venous TSH (vTSH), and free T4) were evaluated according to gestational age, delivery mode, and inferred exposure to povidone-iodine used for cesarean skin preparation. Serum free T4 concentrations were significantly lower in preterm and NICU infants compared with term controls (p = 0.007), while cTSH and vTSH levels were not significantly different. Among NICU infants, those delivered by cesarean section had lower free T4 levels than those born vaginally (p = 0.039), and free T4 was particularly reduced in infants with sepsis (p = 0.022). A moderate correlation between cTSH and vTSH was observed (ρ = 0.50, p < 0.001). These findings indicate that lower free T4 levels in preterm and clinically ill neonates likely represent transient, adaptive responses rather than permanent hypothyroidism, and that differences by delivery mode may reflect a combination of perinatal factors and inferred iodine exposure.

## Introduction

The neonatal intensive care unit (NICU) introduces distinctive challenges for universal newborn thyroid screening programs, which are designed to ensure the early detection and treatment of congenital hypothyroidism (CH). In this setting, both prematurity and concurrent critical illness may alter thyroid function parameters, making interpretation of thyroid function tests (TFTs) particularly complex [1,2]. Preterm infants, whose hypothalamic-pituitary-thyroid (HPT) axis is functionally immature, are especially prone to delayed thyroid-stimulating hormone (TSH) elevation and transient hypothyroxinemia of prematurity (THOP) [3,4]. Moreover, systemic illness and medical interventions commonly encountered in NICU practice may further influence thyroid hormone regulation even in term neonates [5,6].

Povidone-iodine is a widely used and effective perinatal antiseptic; however, significant transcutaneous iodine absorption has been reported, particularly among premature and critically ill infants. Excess iodine exposure can transiently inhibit thyroid hormone synthesis through the acute Wolff-Chaikoff effect, resulting in temporary hypothyroxinemia without a compensatory rise in TSH [7-9]. Recent reports have raised concern about the effects of routine iodine-based antiseptics during delivery on neonatal thyroid function, primarily when confirmatory venous evaluation is not performed [10,11]. These findings suggest that even full-term infants may develop transient thyroid hormone suppression following perinatal iodine exposure.

Newborn screening programs primarily rely on capillary TSH (cTSH) measurement. While this approach has substantially reduced the number of missed CH cases, discrepancies between screening and confirmatory venous TFTs are frequently observed in hospitalized neonates [12]. Such inconsistencies can result in both false-positive and false-negative outcomes, particularly among infants with perinatal iodine exposure or those undergoing hormonal adaptation due to prematurity.

This study aimed to evaluate thyroid function profiles in neonates admitted to the NICU compared with healthy newborns and to investigate potential factors contributing to transient hypothyroxinemia. The relationship between cTSH and venous TSH (vTSH) measurements was analyzed to assess the reliability of screening results. Thyroid hormone levels were further compared according to gestational age (term vs. preterm) and perinatal exposure to povidone-iodine. Finally, the study sought to determine whether the observed reduction in venous fT4 levels, despite normal TSH concentrations, represented a transient suppression of thyroid hormone synthesis rather than primary hypothyroidism. While previous studies focused on either prematurity or illness separately, this is the first study to simultaneously evaluate the combined effects of prematurity, delivery mode, and povidone-iodine exposure on neonatal thyroid function in a Turkish population.

## Materials and methods

Study design and setting

This retrospective, observational study was conducted in the NICU of Memorial Şişli Hospital between January 2023 and June 2025. Of the initially screened neonates, five were excluded: one receiving biotin-containing supplementation (due to potential immunoassay interference), one with a chromosomal abnormality, one with a major congenital anomaly, and two with incomplete medical records. The final analytic sample consisted of 152 NICU infants who met all inclusion criteria and had complete thyroid function data. By mode of delivery, 86.2% (n=131) were born by cesarean section and 13.8% (n=21) by normal spontaneous vaginal delivery. Approximately 20% of the cohort were preterm (27-36 weeks’ gestation); 50% were admitted with transient tachypnea of the newborn (TTN), 10% with sepsis, and 20% with indirect hyperbilirubinemia (jaundice). For comparison, 50 healthy neonates born in the same period and without medical problems served as the control group. The patient group comprised infants admitted within the first week of life who had confirmatory venous TFTs available between postnatal days 10-20 following newborn screening. Infants with chromosomal abnormalities, major congenital malformations, or incomplete medical records were excluded.

Definitions

THOP was defined as low free thyroxine (fT4) in the presence of normal or low TSH. Reference intervals were TSH: 0.73-4.77 mIU/L and fT4: 0.87-1.52 ng/dL.

Data collection and assay standardization

For each infant, gestational age, mode of delivery, and perinatal exposure to povidone-iodine were recorded. Povidone-iodine exposure was defined as the indirect, brief contact resulting from standard maternal skin preparation during cesarean delivery. NICU records confirmed that no infant received direct or repeated povidone-iodine application (e.g., for umbilical care, skin antisepsis, or procedural purposes) after birth. Laboratory data included cTSH, vTSH, and fT4. Capillary samples were obtained by heel prick on days 3-5 of life. Free T3 and reverse T3 were not routinely measured in our institution and were therefore not available for analysis. All venous samples were collected in the morning (08:00-10:00). Infants receiving biotin-containing medications or vitamin supplements before testing were excluded to avoid potential assay interference. Infants receiving biotin-containing medications or vitamin supplements before testing were excluded because biotin can interfere with streptavidin-biotin-based immunoassays, leading to falsely low TSH and falsely high fT4 results.

All measurements were performed in the hospital’s central biochemistry laboratory using the same analyzer and lot numbers throughout (e.g., Abbott Architect i2000SR, chemiluminescence immunoassay; Abbott Laboratories, Abbott Park, IL). Internal quality controls were run according to the manufacturer’s recommendations.

Grouping and subgroup analyses

Infants were classified according to the following:

1. Group: patient (NICU) vs control (healthy).

2. Gestational age: term vs preterm.

3. Perinatal povidone-iodine exposure: exposed (cesarean delivery) vs non-exposed (vaginal delivery using chlorhexidine-based antisepsis).

Prespecified subgroup comparisons were conducted according to this schema. Cases with missing key variables (cTSH, vTSH, or fT4) or without a required confirmatory venous sample during follow-up were excluded from the analyses. The final analytic sample size is reported after all exclusions.

The study protocol was approved by the Memorial Şişli Hospital Ethics Committee (approval no.: 003; date: 22.09.2025). Written informed consent was obtained from the parents of all included neonates. All procedures conformed to the Declaration of Helsinki, and patient confidentiality was strictly maintained.

Statistical analysis

All analyses were performed using IBM SPSS Statistics (version 26.0; IBM Corp., Armonk, NY, USA). The distribution of continuous variables was assessed with the Kolmogorov-Smirnov test. Normally distributed data are presented as mean ± standard deviation (SD), whereas non-normally distributed data are summarized as median (interquartile range (IQR)) or median (min-max), as appropriate.

Between-group comparisons of continuous variables were conducted using the Student’s t-test for normally distributed data or the Mann-Whitney U test for non-normally distributed data. Categorical variables were compared using the chi-square test or Fisher’s exact test when expected cell counts were <5. Correlation between cTSH and vTSH values was evaluated using Spearman’s rank correlation (or Pearson’s correlation when both variables were normally distributed).

All tests were two-tailed, and a p-value <0.05 was considered statistically significant. Where relevant, 95% confidence intervals (95% CI) were reported.

For nonparametric pairwise comparisons (Mann-Whitney U), effect size r was calculated; however, due to the consistent direction and statistical significance of findings, detailed effect size values were not tabulated.

(If multiple subgroup comparisons are performed - for example, across more than two clinical subgroups - overall differences were first assessed with one-way analysis of variance (ANOVA) or Kruskal-Wallis tests, followed by appropriate post-hoc pairwise comparisons.) 

## Results

Among the 152 neonates included in the study, 131 (86.2%) were delivered by cesarean section, and 21 (13.8%) were born via normal spontaneous vaginal delivery. Since povidone-iodine is routinely used for maternal skin antisepsis during cesarean section at our institution, these infants were considered to have experienced brief maternal iodine exposure, whereas those born vaginally were regarded as the non-exposed group.

The control group consisted of 50 healthy neonates. Among them, 38 (76.0%) were delivered by cesarean section and 12 (24.0%) by normal spontaneous vaginal delivery.

**Figure 1:**
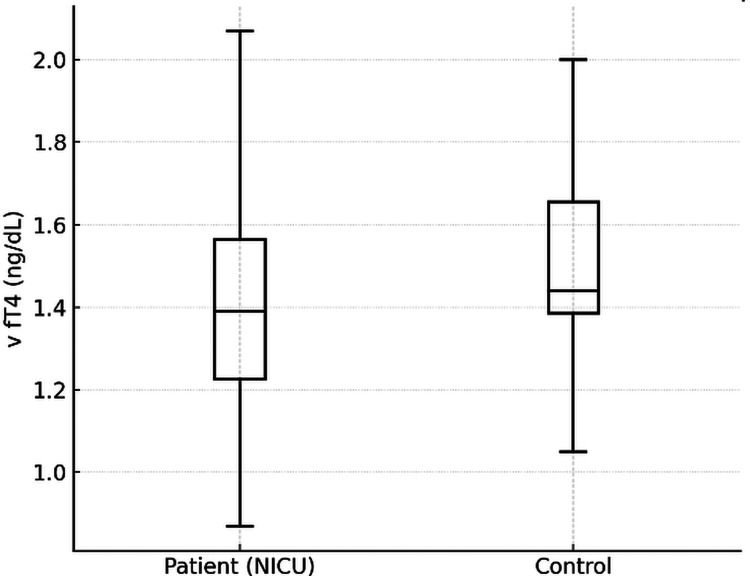
Comparison of v fT4 levels between patient and control group v fT4, venous free T4; NICU, neonatal intensive care unit.

When thyroid function parameters were compared between the NICU and control groups, cTSH and vTSH levels showed no significant difference, whereas venous fT4 concentrations were significantly lower in NICU patients (p = 0.007), suggesting a subtle thyroidal suppression associated with illness or environmental factors during hospitalization (Figure 1, Table 1).

**Table 1:** Comparison of thyroid function parameters between the patient and the control groups TSH, thyroid-stimulating hormone; cTSH, capillary TSH; vTSH, venous TSH; fT4, free thyroxine.

Parameter	Patient (n = 152)	Control (n = 50)	p-Value
cTSH (mIU/L)	2.52 (2.1-2.9)	2.25 (2.1-2.9)	0.664
vTSH (mIU/L)	3.56 (2.9-4.3)	3.88 (3.0-4.6)	0.368
fT4 (ng/dL)	1.42 (1.2-1.6)	1.53 (1.3-1.7)	0.007

Of the 152 neonates included in the study, 78 (51.3%) were preterm, and 74 (48.7%) were term. When thyroid function parameters were compared according to gestational age, there were no significant differences in cTSH or vTSH levels between term and preterm infants (p = 0.317 and p = 0.799, respectively). However, venous fT4 concentrations were significantly lower in preterm neonates (median 0.84 ng/dL) compared with term infants (median 1.48 ng/dL) (p = 0.006). All infants in the control group were term neonates (Table 2).

**Table 2:** Comparison of thyroid function parameters according to gestational age TSH, thyroid-stimulating hormone; cTSH, capillary TSH; vTSH, venous TSH; fT4, free thyroxine.

Parameter	Term (n = 74)	Preterm (n = 78)	p-Value
cTSH (mIU/L)	2.57 (2.1-3.0)	2.46 (2.0-2.8)	0.317
vTSH (mIU/L)	3.46 (2.8-4.0)	3.66 (3.0-4.2)	0.799
fT4 (ng/dL)	1.48 (1.3-1.7)	0.84 (0.7-1.1)	0.006

**Figure 2:**
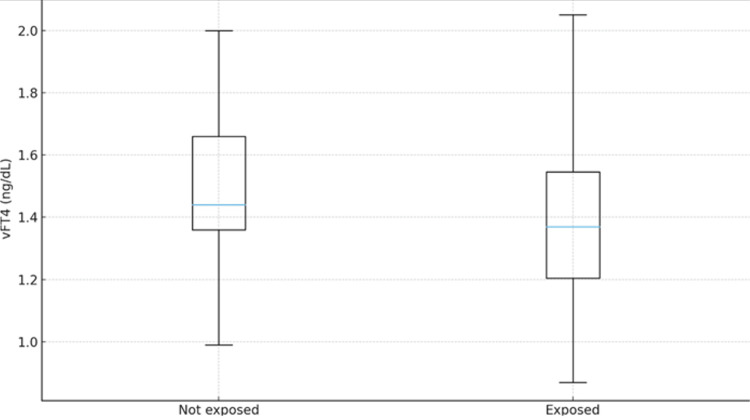
Comparison of v fT4 levels according to povidone-iodine exposure in the patient group Box plots comparing venous fT4 concentrations in NICU patients delivered by cesarean section (exposed to povidone-iodine, n = 131) versus normal spontaneous vaginal delivery (non-exposed, n = 21). Infants exposed to povidone-iodine during cesarean delivery had significantly lower fT4 levels (p = 0.039, Mann-Whitney U test). Box plot elements are as described in Figure 1. v fT4, venous free T4; NICU, neonatal intensive care unit.

Regarding the mode of delivery, neonates born by cesarean section - and therefore exposed to povidone-iodine during maternal skin antisepsis - had significantly lower fT4 levels compared with those born by normal spontaneous vaginal delivery (p = 0.039). No such difference was observed within the control group (Figure 2, Tables 3, 4).

**Table 3:** Comparison according to mode of delivery TSH, thyroid-stimulating hormone; cTSH, capillary TSH; vTSH, venous TSH; fT4, free thyroxine.

Parameter	Cesarean section (n = 131)	Vaginal delivery (n = 21)	p-Value
cTSH (mIU/L)	2.00 (1.8-2.3)	2.70 (2.3-3.1)	0.198
vTSH (mIU/L)	3.00 (2.6-3.4)	2.78 (2.4-3.1)	0.278
fT4 (ng/dL)	1.37 (1.1-1.6)	1.44 (1.2-1.7)	0.039

**Table 4:** Comparison in the control group by delivery mode TSH, thyroid-stimulating hormone; cTSH, capillary TSH; vTSH, venous TSH; fT4, free thyroxine.

Parameter	Cesarean section (n = 38)	Vaginal delivery (n = 12)	p-Value
cTSH (mIU/L)	1.85 (1.5-2.2)	2.20 (1.9-2.5)	0.050
vTSH (mIU/L)	1.95 (1.6-2.4)	3.75 (3.0-4.5)	0.056
fT4 (ng/dL)	1.40 (1.2-1.6)	1.46 (1.3-1.7)	0.140

**Figure 3:**
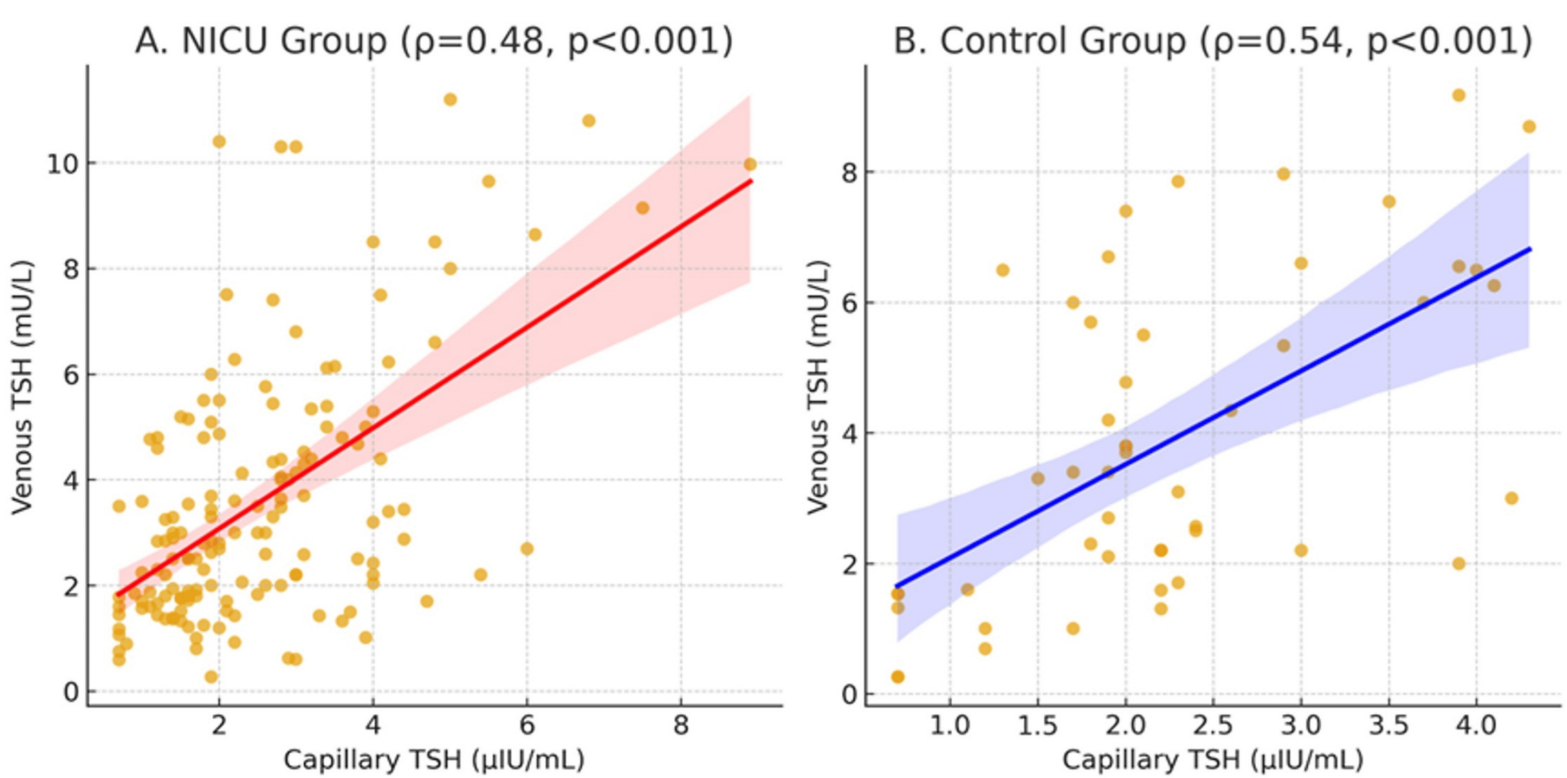
Correlation between capillary and venous TSH levels Scatter plot showing the correlation between cTSH measured on days 3-5 and vTSH measured on days 10-20 in all study participants (N = 202). The solid line represents the line of best fit, and the shaded area represents the 95% confidence interval. A moderate positive correlation was observed (Spearman's ρ = 0.50, p < 0.001). Separate correlations for patient (ρ = 0.48, n = 152) and control (ρ = 0.54, n = 50) groups are shown in different colors/symbols. TSH, thyroid-stimulating hormone; cTSH, capillary TSH; vTSH, venous TSH; NICU, neonatal intensive care unit.

Correlation analysis between cTSH and vTSH demonstrated a significant association in both groups. In the patient group, the correlation was moderate (ρ = 0.48; p < 0.001, N = 152), whereas in the control group, it was slightly stronger (ρ = 0.54; p < 0.001, N = 50) (Figure 3). When all infants were analyzed together, the correlation remained significant (ρ = 0.50; p < 0.001, N = 202), indicating partial concordance between screening and confirmatory venous TSH measurements (Figure 3).

Venous fT4 levels differed significantly among the clinical diagnostic subgroups (Kruskal-Wallis p = 0.018). Post-hoc pairwise comparisons showed that fT4 concentrations were significantly lower in preterm and sepsis groups compared with those with TTN and neonatal jaundice (p = 0.004 and p = 0.022, respectively). No significant difference was observed between the TTN and jaundice groups (p = 0.276). No intergroup differences were found in cTSH or vTSH levels (Table 5).

**Table 5:** Comparison of thyroid function according to clinical diagnosis *p < 0.05, statistically significant difference. TSH, thyroid-stimulating hormone; cTSH, capillary TSH; vTSH, venous TSH; fT4, free thyroxine.

Clinical diagnosis	cTSH (µIU/mL), median (IQR)	vTSH (mU/L), median (IQR)	v fT4 (ng/dL), median (IQR)	p-Value (v fT4)
Transient tachypnea of the newborn (TTN)	2.50 (2.0-2.8)	3.40 (2.6-3.8)	1.30 (1.1-1.5)	0.041
Preterm infant	2.40 (2.0-2.6)	3.60 (2.9-3.9)	0.85 (0.7-1.0)	0.004
Neonatal jaundice	2.60 (2.1-2.9)	3.50 (2.8-3.7)	1.45 (1.3-1.6)	0.276
Neonatal sepsis	1.80 (1.5-2.1)	3.00 (2.3-3.3)	1.10 (0.9-1.3)	0.022

## Discussion

This study aimed to investigate the relationship between thyroid function and key perinatal factors, including mode of delivery, gestational age, and clinical condition at birth. By comparing neonates hospitalized in the NICU with healthy newborns, we sought to determine how prematurity, illness severity, and potential exposure to iodine-containing antiseptics during cesarean delivery may influence thyroid hormone levels and contribute to THOP.

In preterm infants, immaturity of the HPT axis predisposes to early thyroid dysregulation, most notably a delayed rise in TSH and episodes of transient hypothyroidism [2,3]. This developmental vulnerability is further complicated by concurrent systemic illness and common NICU interventions, which can alter thyroid hormone dynamics and make interpretation of TFTs challenging in both preterm and critically ill term neonates [4,5]. Consistent with these observations, in our cohort, serum free T4 levels were found to be lower among preterm infants and those hospitalized in the NICU, supporting the notion that both illness and prematurity contribute to transient hypothyroxinemia. These findings are in line with previous studies discussing the mechanisms and management controversies of transient hypothyroxinemia and delayed TSH elevation in preterm infants.

THOP (low free T4 with normal TSH) is frequently observed in preterm neonates and has been linked to adverse neurodevelopmental outcomes [13,14]. However, the critical free T4 threshold required for optimal brain development and the timing or indication for replacement therapy remain uncertain [15]. It is also suggested that low thyroid hormone levels may represent an adaptive response to illness aimed at reducing metabolic demands [16]. Delayed TSH elevation, another transient form of CH, is also common in premature infants [17]. The benefit of L-T4 (levothyroxine) therapy in such cases remains unclear. Some authors recommend early treatment when TSH exceeds 50 mIU/L because of potential neurodevelopmental risk, whereas others report no significant difference in growth or neurological outcomes compared with untreated controls [2,4]. Moreover, randomized trials in very-low-birth-weight infants with transient hypothyroxinemia (TSH < 10 mIU/L, free T4 < 0.8 ng/dL) have shown that L-T4 supplementation does not improve growth or neurodevelopment at follow-up [18,19].

In our study, the observed pattern of lower free T4 without a corresponding TSH rise parallels these reports. It supports the concept that transient thyroid dysfunction in preterm or critically ill neonates reflects a physiological adaptation rather than true CH. Nevertheless, given the potential long-term neurodevelopmental implications, careful monitoring and individualized follow-up remain essential. The decline in T4 was particularly evident among infants with sepsis, most of whom were treated with ampicillin and gentamicin, suggesting that both systemic inflammation and antibiotic exposure may transiently suppress thyroid hormone synthesis or peripheral metabolism. In line with previous reports, this pattern likely reflects the non-thyroidal illness response associated with systemic inflammation. Dilli and Dilmen also demonstrated that elevated interleukin-6 and C-reactive protein levels were correlated with decreased thyroid hormone concentrations in preterm infants with non-thyroidal illness, supporting the role of inflammatory cytokines in the suppression of thyroid function during sepsis [20]. However, because antibiotic-related effects on thyroid function have not been consistently demonstrated in neonatal populations, this interpretation remains speculative, and we acknowledge this uncertainty.

Previous studies have reported higher cord blood TSH levels in neonates born through vaginal delivery compared with those delivered by cesarean section, attributed to labor stress and transient hypoxia that stimulate TSH secretion and may transiently reduce T4 concentrations [21,22]. In contrast, in our cohort, serum free T4 levels were significantly lower in infants delivered by cesarean section, particularly among those hospitalized in the NICU (p = 0.039). Given that direct iodine measurements were not performed, this association should be interpreted cautiously, and causality related specifically to iodine exposure cannot be confirmed.

Furthermore, beyond iodine exposure, cesarean delivery may influence early thyroid adaptation through other mechanisms, including reduced labor-associated stress responses, altered neonatal transition, and higher NICU admission rates. This finding may be partly explained by the use of povidone-iodine antiseptics during cesarean delivery, which can lead to transcutaneous or mucosal iodine absorption and transient inhibition of thyroid hormone synthesis (the Wolff-Chaikoff effect). Excess iodine exposure in the perinatal period has long been recognized as a transient inhibitor of neonatal thyroid function, particularly in preterm or critically ill infants [7,9,10].

In the control group, cTSH levels were slightly higher in infants delivered vaginally than in those delivered by cesarean section, with borderline statistical significance (p = 0.050). This trend is consistent with previous reports suggesting that labor and delivery stress transiently stimulates TSH secretion in otherwise healthy newborns. In line with physiological expectations, no significant differences in vTSH or venous fT4 levels were observed between delivery modes in the control group, indicating that these transient hormonal variations normalize shortly after birth in healthy infants. Our results on the modest correlation between cTSH and vTSH are consistent with previous reports. Olivieri et al. found only partial concordance between screening and confirmatory thyroid function data in a large cohort of neonates, particularly in those from iodine-deficient regions [10]. Similarly, the recent ESPE consensus guidelines underline that although cTSH is an excellent screening tool, confirmatory venous measurements remain indispensable to establish the diagnosis of CH (4). In our cohort, the correlation between cTSH and vTSH was moderate in NICU patients (ρ = 0.48) and slightly stronger in the control group (ρ = 0.54), suggesting partial, but not perfect, concordance between the two measurements. These findings support current recommendations, which emphasize that screening results should always be interpreted alongside confirmatory venous testing to avoid potential over- or underdiagnosis.

The strengths of our study include a relatively large NICU sample, the inclusion of a healthy control group, and the systematic recording of perinatal variables. However, some limitations must be acknowledged. First, the retrospective and single-center design may limit the generalizability of our findings. Second, thyroid function was not followed longitudinally beyond the neonatal period, which would have allowed a better understanding of the persistence of abnormalities. Third, direct measurement of iodine exposure was not available, which limits the strength of conclusions regarding the relationship between cesarean delivery and thyroid function changes. Fourth, maternal thyroid function data were not available in the electronic records, which is another limitation, as maternal thyroid status may influence neonatal thyroid hormone levels. Finally, potential confounding factors such as illness severity or concomitant medications could not be fully controlled.

## Conclusions

In conclusion, our study demonstrates that while cTSH and vTSH are significantly correlated, individual discrepancies necessitate confirmatory venous testing in selected neonates. Lower fT4 levels in NICU infants and the association with povidone-iodine exposure further highlight the influence of perinatal and iatrogenic factors on neonatal thyroid function. Clinically, low fT4 with normal TSH in preterm or critically ill neonates should prompt repeat testing after stabilization (typically 1-2 weeks later), rather than immediate initiation of L-T4 therapy. Treatment should be considered primarily when TSH becomes persistently elevated or when biochemical abnormalities do not resolve on follow-up. Prospective, multicenter studies with long-term follow-up are needed to better clarify the clinical implications of these findings.
